# 

**Published:** 1857-10

**Authors:** 


					﻿No. V.]
OCTOBER.
[Vol. XIII.
BUFFALO
MEDICAL JOURNAL
AND
jltontljhj Uemew
or
MEDICAL AND SURGICAL SCIENCE.
SANFORD B. HUNT, M. D.,
EID I TO E.
AUSTIN FLINT, Jr., M. D.,
ASSOCIATE EDITOR.
BUFFALO, N. Y.:
E. R. J E WETT <fc CO.’S STEAM PRESS.
Commercial Advertiser Buildings.
1857.
Price $2.00 in advance, or $2.50 at the end of three monthf.
				

